# Supplementary material for: Propofol-based total intravenous anesthesia is associated with less postoperative recurrence than desflurane anesthesia in thyroid cancer surgery
Source: PLoS One. 2024 Jan 5;19(1):e0296169. doi: 10.1371/journal.pone.0296169 (PMC10769032; doi:10.1371/journal.pone.0296169)
Supplement: S1 Table — (DOCX) [file pone.0296169.s001.docx]

**Surgeon Volume (n = 11) in Multivariable Models for Overall Patients (Cox Proportional Hazards Regression for Recurrence)**

| **Variables in the Equation** | | | | | | | | | |
| --- | --- | --- | --- | --- | --- | --- | --- | --- | --- |
|  | B | SE | Wald | df | Sig. | Exp(B) | 95.0% CI for Exp(B) | |  |
|  |  |  |  |  |  |  | Lower | Upper |  |
| Propofol anesthesia (ref: desflurane) | -.734 | .236 | 9.666 | 1 | .002 | .480 | .302 | .762 |  |
| Calendar period (year; ref: 2009-2011) |  |  | 49.015 | 3 | .000 |  |  |  |  |
| Calendar period (2012-2014) | .264 | .360 | .538 | 1 | .463 | 1.302 | .643 | 2.636 |  |
| Calendar period (2015-2017) | .409 | .366 | 1.248 | 1 | .264 | 1.505 | .735 | 3.081 |  |
| Calendar period (2018-2019) | 1.950 | .352 | 30.638 | 1 | .000 | 7.025 | 3.523 | 14.011 |  |
| Charlson comorbidity index | .079 | .064 | 1.534 | 1 | .215 | 1.082 | .955 | 1.226 |  |
| Surgical procedure (ref: total thyroidectomy) |  |  | 65.640 | 2 | .000 |  |  |  |  |
| Lobectomy | .143 | .301 | .225 | 1 | .635 | 1.154 | .640 | 2.081 |  |
| Subtotal thyroidectomy | 1.817 | .229 | 62.907 | 1 | .000 | 6.151 | 3.926 | 9.637 |  |
| pTNM stage of primary tumor, (ref: I) |  |  | 11.426 | 3 | .010 |  |  |  |  |
| II | .904 | .274 | 10.897 | 1 | .001 | 2.470 | 1.444 | 4.224 |  |
| III | .437 | .339 | 1.660 | 1 | .198 | 1.548 | .797 | 3.007 |  |
| IV | .113 | .590 | .037 | 1 | .848 | 1.120 | .353 | 3.557 |  |
| Poor differentiation | -.448 | .501 | .798 | 1 | .372 | .639 | .239 | 1.707 |  |
| Positive margin | .170 | .262 | .423 | 1 | .516 | 1.186 | .710 | 1.981 |  |
| Chemotherapy | -.096 | .689 | .019 | 1 | .889 | .908 | .235 | 3.506 |  |
| Surgeon (ref: Surgeon 1) |  |  | 10.338 | 10 | .411 |  |  |  |  |
| Surgeon 2 | .875 | 1.022 | .733 | 1 | .392 | 2.399 | .323 | 17.788 |  |
| Surgeon 3 | .067 | .243 | .077 | 1 | .782 | 1.070 | .665 | 1.721 |  |
| Surgeon 4 | -13.136 | 1443.378 | .000 | 1 | .993 | .000 | .000 | . |  |
| Surgeon 5 | .821 | .610 | 1.812 | 1 | .178 | 2.273 | .688 | 7.516 |  |
| Surgeon 6 | 1.540 | 1.034 | 2.218 | 1 | .136 | 4.664 | .615 | 35.384 |  |
| Surgeon 7 | .596 | 1.015 | .345 | 1 | .557 | 1.815 | .248 | 13.267 |  |
| Surgeon 8 | -.006 | .329 | .000 | 1 | .986 | .994 | .522 | 1.895 |  |
| Surgeon 9 | -10.542 | 235.194 | .002 | 1 | .964 | .000 | .000 | 4.158E+195 |  |
| Surgeon 10 | 1.741 | .783 | 4.949 | 1 | .026 | 5.705 | 1.230 | 26.455 |  |
| Surgeon 11 | .762 | 1.062 | .515 | 1 | .473 | 2.143 | .267 | 17.193 |  |
