# Supplementary material for: Propofol-based total intravenous anesthesia is associated with less postoperative recurrence than desflurane anesthesia in thyroid cancer surgery
Source: PLoS One. 2024 Jan 5;19(1):e0296169. doi: 10.1371/journal.pone.0296169 (PMC10769032; doi:10.1371/journal.pone.0296169)
Supplement: Attachment — Submitted filename: Reply_template_PONE_R1.docx [file pone.0296169.s003.docx]

**Reply to the reviewers’ comments**

| Reviewer Number | Original comments of the reviewer | Reply by the author(s) | Changes done on page number and line number |
| --- | --- | --- | --- |
| 1 | 1. The low mortality rate in this study, limited by the characteristics of thyroid cancer, may lead to lower statistical efficacy and needs to be mentioned in the limitations. | Thank you for your kind reminder, we have revised the manuscript as you suggested. | P18, Line 3-4. |
| 1 | 2. The addition of some sensitivity analyses could help to enhance the credibility of this study. | We added three subgroup analyses, namely, stratified by sex, hormone therapy and surgical procedure as the alternatives to sensitivity analyses for enhancing the credibility of this study (Table 3). | Table 3. |
| 2 | 1. STROBE Statement. Considering as a cohort study, it is better for authors to check all the necessary elements according to the STROBE checklist provided guidance on how to report observational research well, which could achieved on https://www.strobe-statement.org/. | Thank you for your kind reminder, we have revised the manuscript according to the necessary elements of the STROBE checklist. |  |
| 2 | 2. Eligible criteria. Why were the patients with ASA score of I not included in the inclusion criteria? It seems to be unusual that all patients with an average age of 45 years undergoing thyroid surgery within ten years had comorbidities preoperatively. It is better to explain why the patients with ASA I were excluded. Furthermore, other factors which may affect immune function should be considered before enrolling cases, such as the use of nerve block, hormone and dexmedetomidine, since they may also interfere with postoperative recurrence by affecting the immune response. | 1. Cancer is a systemic disease by definition. Weight loss, anemia, previous chemotherapy, or a decrease in performance status should impose a higher ASA score. (J Korean Med Sci. 2017;32:1211-1212.)Therefore, in our hospital, we consider the cancer patient was above ASA I.  2. In our clinical practice, there is no routine use of nerve block or dexmedetomidine for patients under thyroid cancer surgery. On the other hand, we added postoperative hormone therapy in the statistical analyses in this study (Table 1 and 2). | Table 1 and 2. |
| 2 | 3. Surgical method. Surgical method is the most direct factor for postoperative recurrence. Subtotal thyroidectomy is one of the PTC recurrence risk factors (PMID: 25337182). Incomplete tumor removal is also one of the risk factors (PMID: 34102860). However, in this study, this key factor seems to be missed. Moreover, the condition of secondary surgery during the study period should be considered. | We added surgical method (surgical procedure) and incomplete tumor removal (positive margins) in the statistical analyses in this study (Table 1-3). In this study, most patients received the secondary surgery due to postoperative recurrence, therefore, secondary surgery seemed not to be the predictor of recurrence. | Table 1-3. |
| 2 | 4. Other risk factors for recurrence. Without postoperative 131-I treatment may also be one of the PTC recurrence risk factors (PMID: 25337182). In addition, poor dedifferentiation of the primary tumor is a predictive factor of PTC recurrence (PMID: 23790258). It is better to consider these factors when matching. | Thank you for your kind reminder. We conduct PS matching including those variables which information can be known before operation to mimic RCT. Postoperative 131-I treatment and poor dedifferentiation of the primary tumor were not preoperative factors. Therefore, we analyzed postoperative 131-I treatment and poor differentiation in the Table 2. | Table 2. |
| 2 | 5. Follow-up for postoperative recurrence. The authors did not specify how they collected the data of the recurrence. | We collected the data of the recurrence by the clinical data support of the Cancer Registry Group of TSGH based on patients who developed any evidence of recurrence (such as abnormal sonographic and cytology findings, thyroglobulin levels, and iodine uptake beyond the thyroid bed in a diagnostic whole-body scan) after achieving an excellent response to the primary surgery. (Cancers. 2021;13:5422.) | P11, Line 18-22. |
| 3 | 1. The authors have applied Cox proportional hazards regression analysis for recurrence events. However, given that death events could be a competing risk, wouldn't it be more appropriate to apply the Fine-Gray hazard regression model? | Thank you very much for your valuable opinion. Competing risks analysis refers to a special type of survival analysis whose purpose is to correctly estimate the marginal probability of an event in the presence of competing events. We examined our data set using the Fine-Gray model (cmprsk for R software) and found that competing risks of recurrence are unlikely. Detailed description follows. Among 1209 patients, 47 died before recurrence, with a mortality rate of 3.89%. Compared with the desflurane group, the point estimates of recurrence HR in the propofol group were both 0.385, and the 95% CIs were also very close. (see below)  1.Cox model:  HR (95% CI): 0.385 (0.258, 0.572); P-value: 2.47*10^-6 (< 0.001).  2.Fine-Gray model:  HR (95% CI): 0.385 (0.259, 0.574); P-value: 2.75*10^-6 (< 0.001). |  |
| 3 | 2. It seems that the authors did not include variables like surgeon volume, calendar period, and functional status in the propensity score estimation. When estimating the propensity score model, factors that strongly influence the outcome should be prioritized, and collinearity between covariates is generally not a major issue. Therefore, it would be desirable to include these variables in the propensity score model. | Thank you for this useful suggestion. We have re-run PSM including those variables which information can be known before operation to mimic RCT. The variables were calendar period, sex, age, CCI, functional status, ASA score, tumor size, tumor number, and surgical procedure, except surgeon volume due to it cannot converge in the estimation. Alternatively, we analyzed surgeon volume (n = 11) in multivariable models for overall patients (Cox proportional hazards regression for recurrence), and surgeon volume seemed not to affect recurrence (P = 0.411; Table 2and S1 Table). In addition, since the SMD for calendar period and surgical procedure was greater than 0.1, these variables, as well as surgeon volume, were included in the modeling. (Table 3) | Table 1-3, S1 Table. |
| 3 | 3. For Table 1, it appears that the authors have not calculated the Absolute Standardized Mean Difference (ASMD) for categorical variables. However, Austin & Stuart 2015 (DOI: 10.1002/sim.6607) provides a method for calculating ASMD for categorical variables, which you may find useful. Also, p-values, which are dependent on sample size, are not sufficient evidence to indicate the magnitude of differences between groups, so they could be omitted. | We have added the SMD in the Table 1 where ever it is necessary. | Table 1. |
| 3 | 4. Table 2 presents the results evaluating the association between each covariate and the outcome. However, since the primary objective of this paper is to evaluate the association between the type of anesthesia and the outcome, I do not see significant relevance in this examination. | Thank you for your friendly advice. Because other reviewers suggest that we shall provide additional data analysis in the Table 2, we prefer to keep Table 2 in this study. | Table 2. |
| 3 | 5. Table 3 only presents the results showing the relationship between the type of anesthesia and recurrence. Even if there was no significance, it is necessary to also include the intergroup differences in mortality. | We have revised the manuscript as you suggested. | Table 3. |
| 3 | 6. For Figure 2, the Kaplan-Meier plot, it is necessary to specify what kind of test was used to calculate the p-values. | Log-rank test was used to calculate the P-values. | Figure 2. |
